# Oral Administered Particulate Yeast-Derived Glucan Promotes Hepatitis B Virus Clearance in a Hydrodynamic Injection Mouse Model

**DOI:** 10.1371/journal.pone.0123559

**Published:** 2015-04-09

**Authors:** Xiaoyu Yu, Dandan Zhang, Bisheng Shi, Guangxu Ren, Xiuhua Peng, Zhong Fang, Maya Kozlowski, Xiaohui Zhou, Xiaonan Zhang, Min Wu, Cong Wang, Zhenghong Yuan

**Affiliations:** 1 Shanghai Public Health Clinical Center, Fudan University, Shanghai, China; 2 Key Laboratory of Medical Molecular Virology, School of Basic Medical Sciences, Shanghai Medical College of Fudan University, Shanghai, China; 3 Department of Biochemistry, Microbiology, and Immunology, University of Ottawa, Ottawa, Ontario, K1H 8M5, Canada; Indiana University, UNITED STATES

## Abstract

Hepatitis B virus (HBV) persistent infection is associated with ineffective immune response for the clearance of virus. Immunomodulators represent an important class of therapeutics, which potentially could be beneficial for the treatment of HBV infection. The particulate yeast-derived glucan (PYDG) has been shown to enhance the innate and adaptive immune responses. We therefore, assessed the efficacy of PYDG in enhancing HBV specific immune responses by employing the hydrodynamic injection-based (HDI) HBV transfection mouse model. Mice were intragatric administered PYDG daily for 9 weeks post pAAV/HBV1.2 hydrodynamic injection. PYDG treatment significantly promoted HBV DNA clearance and production of HBsAb compared to control mice. PYDG treatment resulted in recruitment of macrophages, dendritic cells (DCs) and effector T cells to the liver microenvironment, accompanied by a significantly augmented DCs maturation and HBV-specific IFN-γ and TNF-α production by T cell. In addition, enhanced production of Th1 cytokines in liver tissue interstitial fluid (TIF) was associated with PYDG administration. Live imaging showed the accumulation of PYDG in the mouse liver. Our results demonstrate that PYDG treatment significantly enhances HBV-specific Th1 immune responses, accompanied by clearance of HBV DNA, and therefore holds promise for further development of therapeutics against chronic hepatitis B.

## Introduction

Chronic hepatitis B virus (HBV) infection leads to development of liver cirrhosis and hepatocellular carcinoma resulting in severe morbidity and mortality [1]. Currently, chronic HBV infections are treated with nucleos(t)ide analogues and pegylated interferon-alpha (IFN-α). The IFN-α treatment is associated with low efficacy, while nucleos(t)ide analogues cannot completely cure the infection and long-term treatment leads to the drug-resistance [2]. Chronic HBV infections are characterized by virus-induced immune tolerance leaving the host unable to resolve the infection [3–5]. Thus, the effective HBV therapy requires elicitation of a potent anti-HBV immune response and elimination of the viral immunosuppressive factors. Recent promise in inducing prolonged suppression of viral replication following Toll-like receptor 7 (TLR7) agonist treatment of chronically HBV-infected woodchucks and chimpanzees ignited an interest in using immunomodulators as effective stimulants of innate immunity [6]. More importantly, it has also been shown that PYDG treatment could potently induce antitumor immunity resulting in reduced tumor burden [7,8]. However, not all glucan formulations exhibit the same biological activity. For example, soluble glucan primes innate neutrophils via complement and CR3-dependent pathways, while particulate yeast-derived glucan (PYDG) could induce Th1 bias and CTL immune responses [9]. Although glucan has been shown to effectively stimulate innate immune response in animal models of viral infection [10,11], there is no information regarding the use of PYDG for treatment of human hepadnavirus infection. In this study, we employed the HBV HDI-murine model to examine PYDG efficacy in boosting the immune response. We also provide evidence demonstrating a benefit of PYDG treatment in promoting a strong immune response against HBV, resulting in an accelerated HBV DNA clearance without causing liver damage in mice. Collectively, our results suggest that PYDG is an effective immunomodulator, which may be considered for development of new complementing therapeutic interventions against chronic HBV infection.

## Materials and Methods

### Reagents

Highly purified particulate yeast-drived β-glucan from Saccharomyces cerevisiae was kindly provided by BiotecPharmacon (Tromso, Norway). Endotoxin level was lower than 0.05 EU/ml. For the *in vivo* imaging assay, PYDG was labeled with fluorescein dichlorotriazine (DTAF; Molecular Probes, Invitrogen) as described previously [9]. Fluorochrome-conjugated anti-mouse and anti-human monoclonal antibodies (mAbs) for flow cytometry were purchased from eBiosciences. A set of 24 overlapping peptides (18 amino acids in length, overlapping by 10 amino acids, purity >75%) covering the precore and core regions (aa 29–149) of HBV (genotype A, B, C and D) was synthesized by ChinaPeptides Co., Ltd. The region of the core protein containing arginine-rich domain (aa 150–185) was excluded from this study.

### Ethics statement

This study was reviewed and approved by the Ethics Committee of Shanghai Public Health Clinical Center (SHAPHC). No mice died before the end point of experiments. All the mice were sacrifice with CO2 inhalation and efforts were made to minimize suffering.

### HDI-based HBV mouse model and PYDG treatment

Five to six weeks old C57BL/6 male mice were purchased from B&K Universal Group Limited (Shanghai, China) and kept in pathogen-free (SPF) lab at the Center of Laboratory Animals, Shanghai Public Health Clinical Center. The hydrodynamic injection-based HBV mouse model was established as described by Huang et al [12]. Briefly, ten micrograms of plasmid pAAV-HBV1.2 in 2mL PBS was injected into the tail veins of mice within 6 seconds. HBsAg and HBV-DNA level in the plasma was evaluated 3 days post-injection. Mice which tested positive for HBsAg and HBV DNA were divided into two groups, namely the PYDG-treated and the PBS-treated control. PYDG (200 ug in 200 uL of PBS) was delivered by intragastric administration to the PYDG group mice daily for 9 weeks. Blood was collected every week and plasma was isolated and cryopreserved at -80°C until analyzed. HBsAg and HBsAb levels were monitored by chemiluminescent microparticle immunoassay (Abbott Architect). HBV-DNA load was measured by Quantitative Diagnostic Kit (PCR-Fluorescent probing, QIAGEN). ALT and AST levels were detected by Roche Modular P800 Automatic Chemistry Analyzer. At 10 weeks post intragastric administration, the mice were sacrificed and the liver infiltrating lymphocytes and cytokine levels in liver tissue interstitial fluid (TIF) were analyzed by flow cytometry. All mouse experiments were performed in accordance with the Animal Care and Use Committee at Fudan University

### Isolation of hepatic lymphocytes from mouse

Hepatic lymphocytes were isolated from the liver as described previously [13] with some modifications. Briefly, livers were perfused with pre-warmed PBS to flush blood from the hepatic vasculature and were forced through a 70 mm nylon cell strainer (BD Falcon, Franklin Lakes, NJ). After washing, cell pellets were suspended in 5 ml of pre-warmed Ca2^+^/Mg2^+^-free HBSS supplemented with 10% FBS, 0.05% Collagenase type II (Sigma) and 500 U/ml DNAase type I (Sigma) and digested at 37°C for 30 minutes. Cells were then layered onto 40% Percoll solution (Axis-Shield) in RPMI 1640 for density separation, and centrifuged at 2000 g for 15 minutes at 4°C without brakes. Cell yields and viabilities were determined by trypan blue exclusion microscopy.

### TIF (tissue interstitial fluid) collection

The TIF collection protocol was modified according to the Celis’ method [14]. Small 1–3 mm^3^ pieces of fresh liver tissue suspended in PBS were transferred to 50 mL tubes and extensively washed with PBS twice. The samples were then centrifuged at 1000 g for 3 minutes and the supernatant was removed. Samples were further centrifuged at 2000 g for 8 minutes and then at 20000 g for 30 minutes at 4°C. The TIF samples were snap-frozen in liquid nitrogen and stored at -80°C till use.

### IHC staining for HBcAg

HBcAg was visualized by immunohistochemical (IHC). Paraffin-embedded, 6-mm-thick liver sections were stained with rabbit anti–hepatitis B core protein (anti-HBcAg) antibody (Maixin-Bio., China) and visualized using a diaminobenzidine peroxidase substrate kit (Maixin-Bio., China).

### *In vivo* Imaging

To obtain DTAF-PYDG, the DTAF 100 μl (20 mg/ml) was added into 1ml PYDG (10mg/ml) and incubated for 1 hour at room temperature with continuous stirring. Mice were orally administered with 200ug/200ul DTAF-PYDG daily for 3 days. 4 hours after the last delivery, mice were anesthetized and visualized under the whole-body optical imaging system to monitor the localization of PYDG.

### Analysis of Immune cells phenotype and functions

The mouse livers were perfused and the infiltrating lymphocytes were isolated. The cells were then stained with either anti-CD4, anti-CD8, anti-CD11C or anti-F4/80 antibodies for analysis of T cells, DCs and macrophages by flow cytometry, respectively. Meanwhile, liver lymphocytes were stimulated with the peptide pools covering HBV the precore/core regions (Genotype A) for 5 hours and then stained with corresponding surface antibodies for 30 minutes at 4°C. For intracellular cytokine staining (ICS), the cells were fixed and permeabilized with fixation/permeabilization solution (BD Biosciences) for 20 minutes at 4°C. After washing the cells once with perm/wash buffer (BD Biosciences), antibodies against IFN-γ, TNF-α and IL-2 were added and the cells were washed again for 30 minutes followed by flow cytometry analyses (BD FACS Aria II).

### Statistical analysis

Results were compared using the nonparametric Kruskal-Wallis test, the nonparametric Mann-Whitney test, or the paired t-test using Prism software (Graphpad, SanDiego, CA). P-values < 0.05 were considered to be significant.

## Results

### Orally administrated PYDG promotes HBV DNA clearance without causing liver inflammation in the HBV transfection mouse model

**Fig 1:**
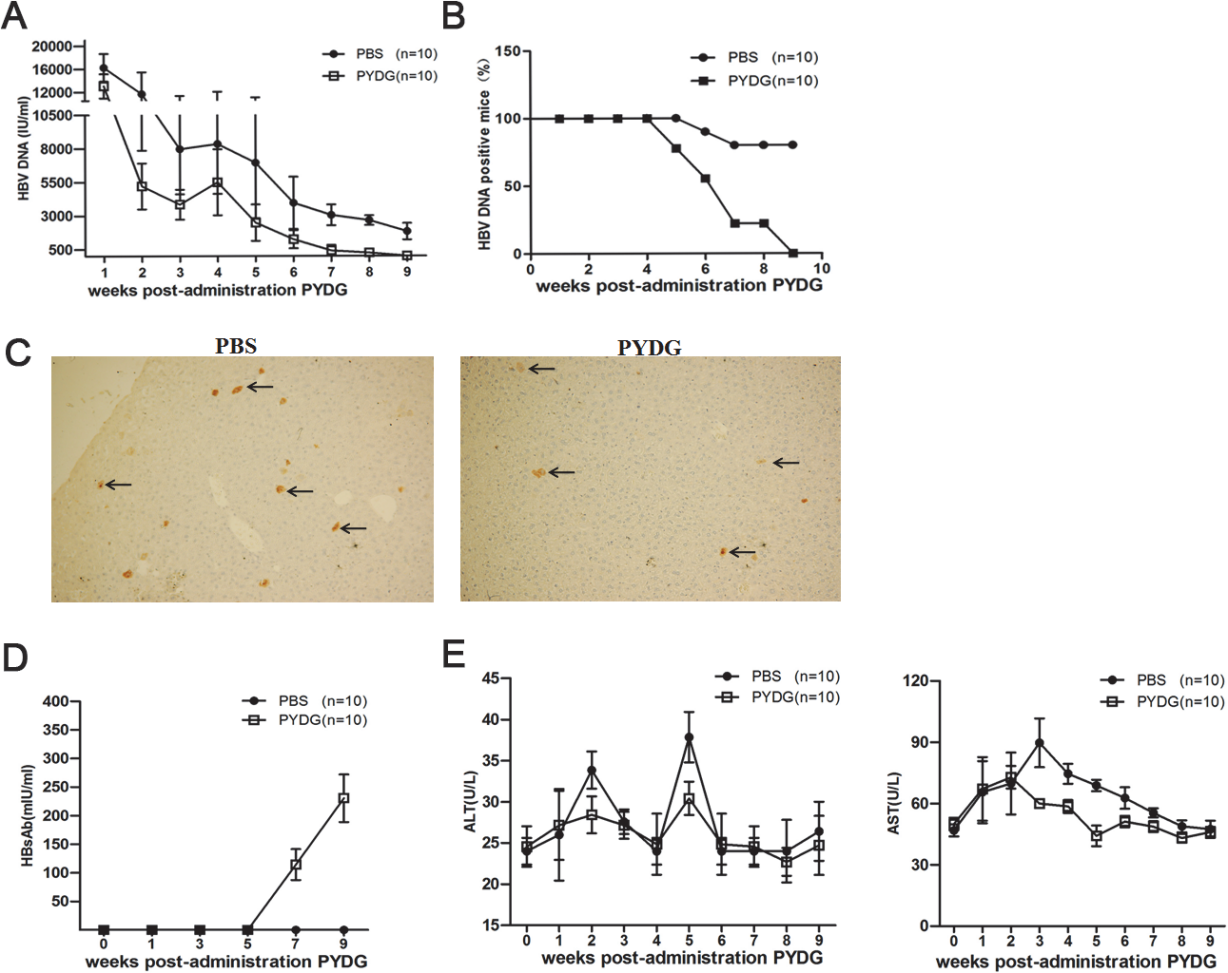
Orally administrated PYDG promotes HBV DNA clearance without causing liver inflammation in the HBV mouse model. HBV-HDI mice were treated orally with PBS or with 200μg PYDG in 200μl PBS (n = 10) daily for 9 weeks. (A) HBV DNA was monitored weekly. (B) Percentage of HBV DNA positive mice over 9 weeks. (C) HBcAg positive hepatocytes were visualized by immunohistochemistry using anti-HBcAg-HRP, arrows indicate HBcAg. (D) The level of HBsAb was measured in the plasma of mice every 2 weeks. (E) ALT and AST levels in plasma of HDI-HBV mice treated with PYDG or PBS were determined over 9 weeks (*, p<0.05; **, p<0.01; ***, p<0.001).

To determine the immunomodulatory effects of PYDG in HBV clearance, levels of HBV DNA and HBsAg in plasma of PYDG-treated and PBS-treated control HDI-HBV mice were monitored every week. PYDG treatment had a significant effect on HBV DNA level, which declined over the course of the experiment and was consistently lower compared to controls (Fig 1A). PYDG-treated mice demonstrated 100% (10/10) of HBV DNA clearance at 9 weeks post treatment while only 30% (3/10) of mice in the control group lost HBV DNA at the same time point (Fig 1B).

Immunohistochemistry staining of the liver tissue under high performance (HP) showed fewer number of HBcAg positive hepatocytes from PYDG-treated compared to PBS-treated mice (with on average 3 cells/HP in PYDG-treated mice compared to 6 cells/HP in PBS-treated mice) (Fig 1C). However, the PYDG treatment had no effect on the decreasing trend of HBsAg between the two groups of mice (S1 Fig). These data suggested that PYDG could effectively accelerate HBV DNA clearance in HBV-infected mice. In addition, HBsAbs (88.33±20.70 mIU/ml) were detectable at week 7 of PYDG-treatment (Fig 1D), demonstrating that PYDG induced specific immune responses against HBV. The murine plasma ALT and AST levels were also measured. The results showed that the ALT and AST levels were not higher in PYDG-treated mice than in PBS-treated controls. At the same time, the AST levels were much lower 3 weeks after PYDG treatment compared to controls (Fig 1E), suggesting that PYDG-treatment did not induce severe liver inflammation.

### PYDG enhances dendritic cells (DCs) maturation and HBV specific Th1 immune response in livers of the HDI- HBV-mice

**Fig 2:**
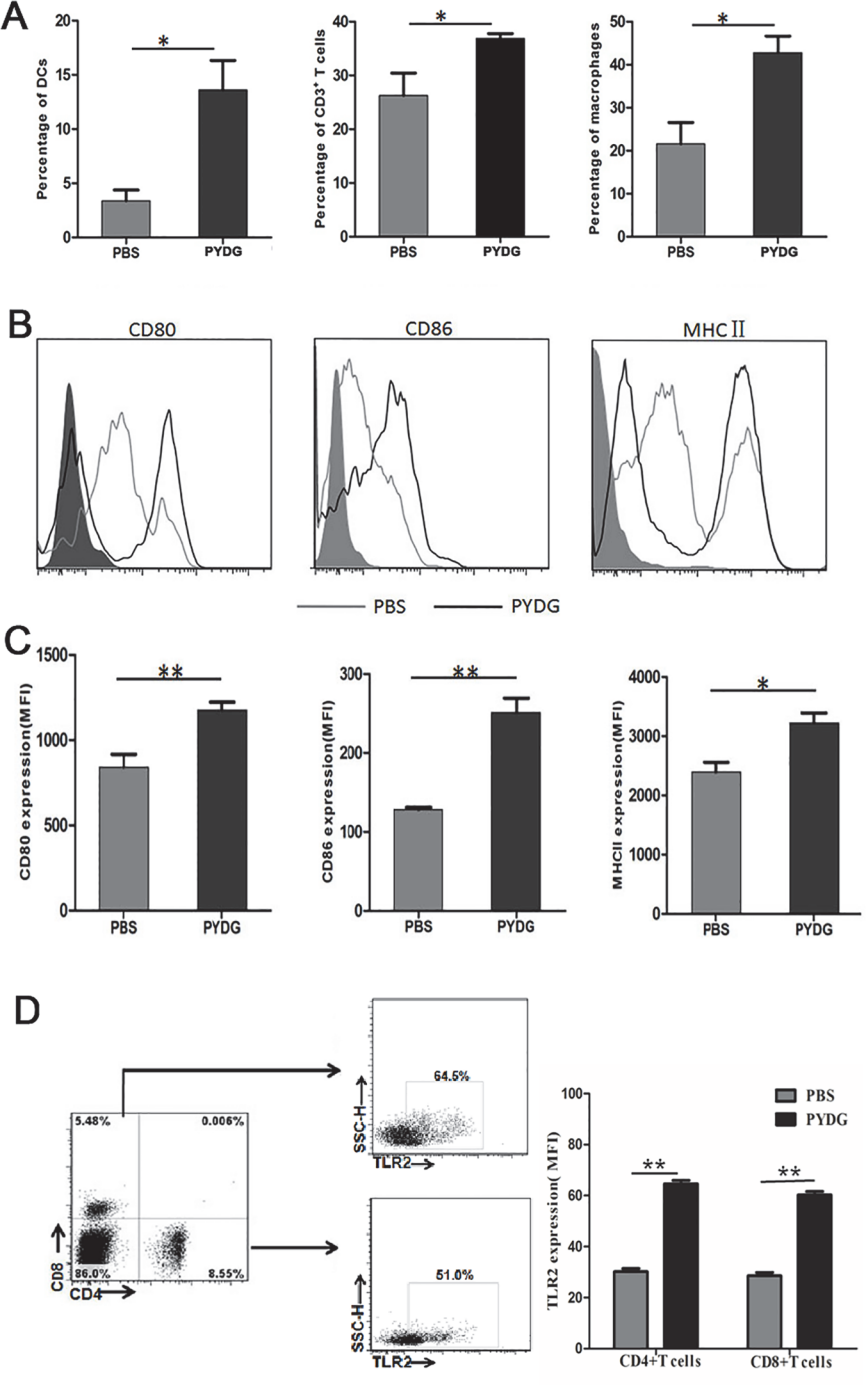
PYDG enhances dendritic cells (DCs) maturation and T cells activation in livers of the HDI-HBV mice. Mice were treated orally with PBS or with 200 μg of PYDG in 200μl PBS daily for 9 weeks and then sacrificed. (A) Expression of CD11c, CD3, F4/80 in liver infiltrating DCs, T cells and macrophages was examined by flow cytometry, left panel, middle panel and right panel, respectively. (B and C) Expression of CD80, CD86 and MHC Ⅱ on infiltrated DCs analyzed by flow cytometry. (D) Expression of TLR2 on CD4^+^ and CD8^+^ T cells analyzed by flow cytometry (*, p<0.05; **, p<0.01; ***, p<0.001).

**Fig 3:**
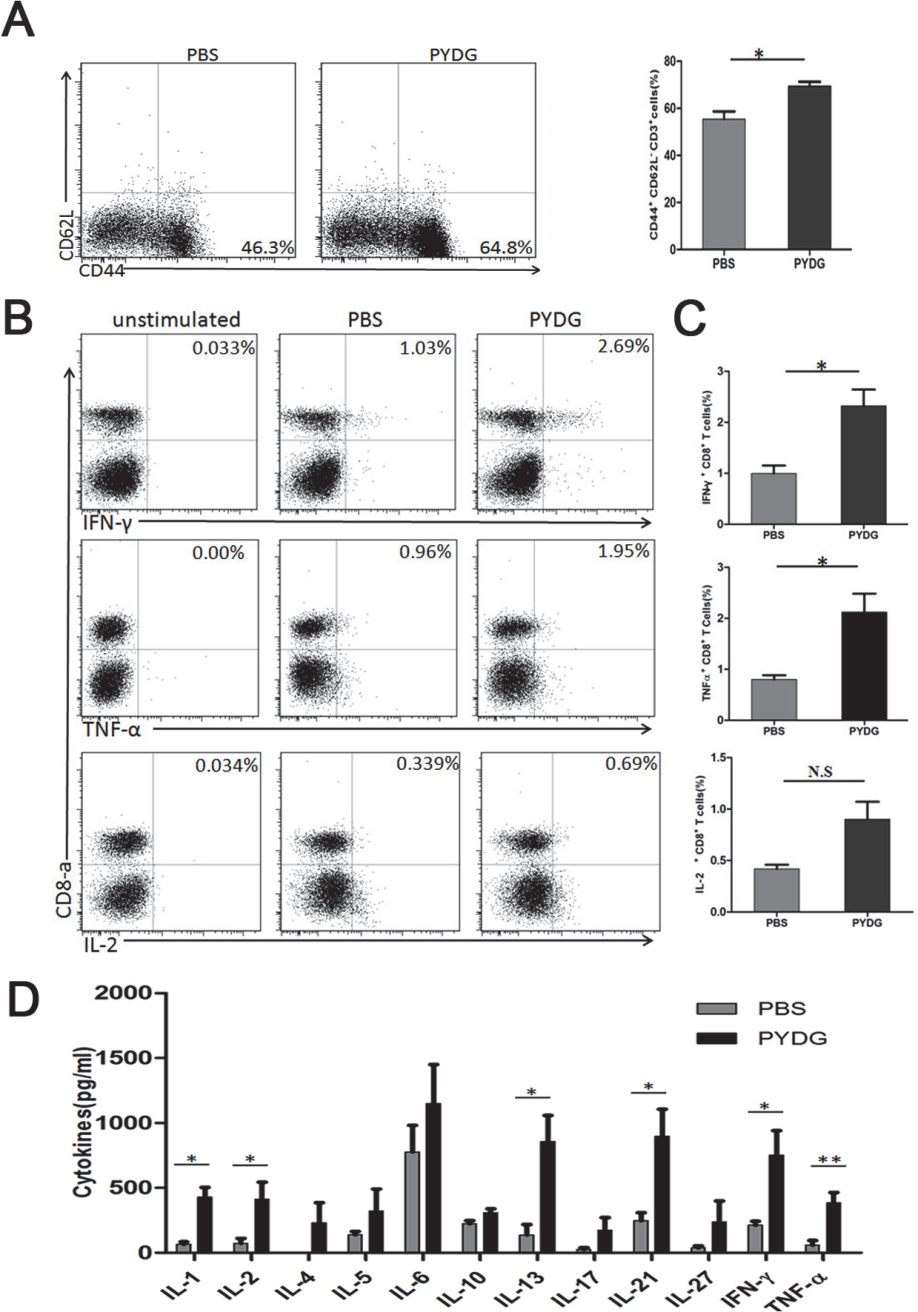
PYDG enhances HBV specific Th1 immune response in livers of the HDI- HBV-mice. Mice were treated orally with PBS or 200μg PYDG daily for 9 weeks. Mice were then sacrificed and isolated liver lymphocytes were stained with mAbs. (A) CD62L^low^CD44^high^ cells were gated on CD3^+^ cells. Proliferation of cells was determined by percentage of CD62L^low^CD44^high^ cells. (B and C) Liver lymphocytes from PYDG-treated and control mice were stimulated with HBV OLP with or without 200 μg/ml PYDG for 5 hours. CD4^+^ and CD8^+^ T cells were then stained for intracellular TNF-α, IFN-γ and IL-2 production and examined by flow cytometry. (D). Liver tissue interstitialfluid (TIF) was extracted from mice treated with or without PYDG (n = 8) and production of cytokines was determined by flowcytomix (*, p<0.05; **, p<0.01; ***, p<0.001).

Antigen presenting cells and T cells are critical for effective HBV immune clearance. To get insights into the mechanisms of PYDG-mediated HBV clearance, the phenotype and function of liver infiltrating T cells, macrophages and DCs were analyzed. HDI-HBV-mice treated with PYDG or PBS were sacrificed at week 9 post-PYDG treatment. The results demonstrated that the percentages of liver infiltrating T lymphocytes were significantly higher in PYDG-treated mice compared to controls (36.93±1.71% vs.26.28±3.38%, P<0.05. Similar effect was observed for macrophages (42.83±7.66% vs. 21.6±6.96, P<0.05) and DCs (13.62±4.43% vs. 3.395±1.94, P<0.05) (Fig 2A), suggesting that PYDG administration attracts immune cells to the liver. Next, we analyzed the effect of PYDG on the maturation of DCs in liver tissue of HDI-HBV mice. For this, we compared the expression of co-stimulatory molecules in PYDG-treated or PBS-treated mice. Results showed that CD86 (MFI: 251.34±31.20 vs. 128.33±4.73, P<0.01), CD80 (MFI: 1177±79.38 vs. 842.33±79.63, P<0.01) and MHC II (MFI: 3222.23±194.07 vs.2394.67±182.34, P<0.05) were significantly up-regulated in PYDG-treated group compared to the control group (Fig 2B and 2C). As CD4+ and CD8+ T cells are crucial for the resolution of HBV infection, we further examined whether DCs activation induced by PYDG could influence the HBV-specific T-cells response. Interestingly, the TLR2, which is a T cell activation co-stimulatory receptor expressed on CD4+ and CD8+ T cells, was strongly up-regulated in PYDG-treated mice. Results showed that TLR2 expression on CD4^+^ T cells (MFI: 30.21±3.20% vs. 64.70± 3.43%, P<0.01), and CD8^+^ T cells (MFI: 27.70%±3.46 vs. 60.31±3.29%, P<0.01) was significantly up-regulated in PYDG-treated group compared to the control group (Fig 2D). In addition, a higher percentage of CD62L^low^CD44^high^ T cells was observed in the livers of PYDG-treated mice compared to controls (69.53±3.44% vs. 55.4±6.49%, P<0.05) (Fig 3A), indicating that PYDG treatment led to an increase in the numbers of effector T cells in livers of HBV-infected mice.

Next, we studied if these HBV-specific effector T-cells were functional and could produce antiviral cytokines such as IFN-γ and TNF-α. Liver-infiltrating lymphocytes were stimulated with HBV overlapping peptides pool (OLP) for 5 hours, intracellularly stained with corresponding cytokines and analyzed by flow cytometry. Results showed that the percentage of IFN-γ^+^ CD8^+^ T cells (2.32±0.44% vs. 0.99±0.11%, P<0.05) and TNF-α^+^ CD8^+^T cells (0. 91±0.19% vs. 0.42±0.07%, P<0.05) were significantly higher following PYDG treatment (Fig 3B and 3C). Similar results were observed in CD4^+^ T cells (data not shown). These data strongly suggest that PYDG treatment leads to an enhanced T cell response against HBV *in vivo*.

In view of the above results, it was of interest to determine whether PYDG-boosted immune responses against HBV could alter the cytokine profiles in the liver milieu. Liver tissue interstitial fluid (TIF) forms the interface between circulating body fluids and intracellular fluids, reflecting the physiological and pathological state of the liver [15]. The cytokine expression profile in TIF from HDI-HBV mice treated with PYDG for 9 weeks was then examined to determine if PYDG treatment induced an anti-viral response in the liver. Results showed that Th1-cytokines, including IL-2, TNF-α and IFN-γ, were significantly increased after PYDG-treatment compared to controls: IL-2 (73.29±43.60 pg/ml vs. 443.62±212.58 pg/ml, P<0.05), TNF-α (34.71±24.38 pg/ml vs. 384.31±87 pg/ml, p<0.01) and IFN-γ (227.34±62.74 pg/ml vs. 618.65±231.24 pg/ml, P<0.05) (Fig 3D). These data suggest that PYDG is efficiently transported into the liver, where it activates liver-associated immune cells to induce Th1 cytokines.

### PYDG accumulates in the mouse liver where it interacts with immune cells

**Fig 4:**
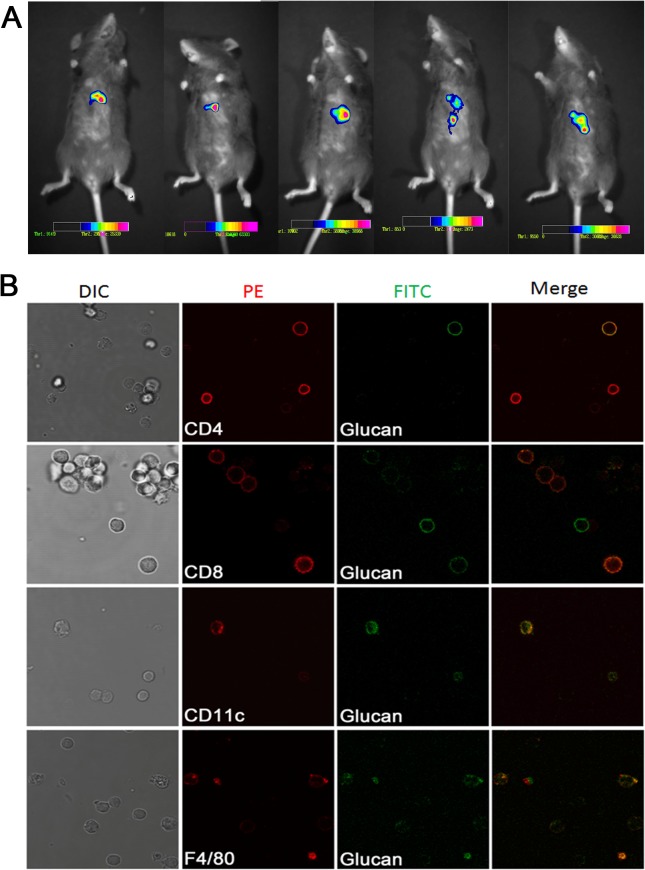
PYDG accumulates in the mouse liver where it interacts with immune cells. DTAF-labeled PYDG was orally administered into C57BL/6 mice (n = 5) for three days. (A) Mice were anesthetized and imaged for the distribution of DTAF-PYDG in liver using fluorescence imaging. (B) Mice were sacrificed and liver lymphocytes were stained with anti-CD11c, anti-F4/80, anti-CD4 and anti-CD8 mAbs. Co-localization of DTAF-PYDG (green) with DCs (CD11c, red), macrophage (F4/80, red), CD4^+^ T (CD4, red), CD8^+^ T (CD8, red) was visualized under microscopy.

To further investigate if PYDG mediates its effects by specifically localizing to the livers of HBV-infected mice, we monitored the localization of PYDG *in vivo*. Mice were fed with DTAF-labeled PYDG for 3 days and PYDG localization was monitored via live imaging with green fluorescence. We observed a localization of PYDG, depicted as bright points, in the liver of PYDG-treated mice but not in the PBS-treated mice (Fig 4A). To examine whether PYDG distributed exclusively to the liver, we also visualized localization of the fluorescence labeled PYDG in frozen section from kidney and intestine under microscopy. Our results demonstrated that PYDG localized to the liver but not to the kidney, suggesting the specificity of the PYDG particles in the liver (S2 Fig).

In view of our results demonstrating that PYDG treatment leads to infiltration of immune cells to the liver, we further studied if there was a direct interaction between PYDG and various subpopulations of liver-associated immune cells. Three days post oral administration of DTAF-labeled PYDG, C57BL/6 mice were sacrificed and liver nonparenchymal cells were isolated and differentially stained with antibodies specific for T cells, macrophages, DCs and examined under confocal microscopy. Results showed that DTAF-labeled PYDG colocalized with CD4^+^, CD8^+^ T cells, CD11c^+^ DCs and F4/80^+^ macrophages with some PYDG particles phagocytosed by CD11c^+^ DCs and F4/80^+^ macrophages (Fig 4B), suggesting that PYDG could internalized by immune cells.

## Discussion

Efforts to treat HBV infection have largely focused on reversing HBV-mediated immune suppression of the liver microenvironment by inducing strong HBV-specific immune response. Particularly, immunomodulators such as agonists of TLRs (TLR2, TLR3, TLR7) have been explored as promising novel interventions in treatment of chronic HBV infection [16,17]. Previously, we have provided the evidence that heat-inactivated recombinant Hansenulapolymorpha yeasts expressing hepatitis B surface antigen (yeast-HBsAg) could promote DCs maturation and enhance HBsAg-specific Th1 and Th2 immune responses in mice [18]. In the present study, employing the HBV-HDI mouse model, we demonstrated that PYDG exhibits potent antiviral and immunostimulatory effects in hepadnaviral infection.

Although an impairment of the innate immune response contributes to the establishment of chronic disease, the hallmark of chronic HBV infection is the lack of a robust HBV-specific CD8^+^ and CD4^+^ T cell response required for efficient HBV elimination [19]. T cell responses are weak or undetectable in chronic HBV patients and reconstitution of CD4+ and CD8+ T cells functions is essential for HBV immunotherapy [20]. Our results demonstrated that PYDG treatment induced the migration of CD62L^low^CD44^high^ T cells into the livers of HBV-HDI mice, enhanced production of IFN-γ and TNF-α and exhibited a robust HBV-specific CD8^+^ and CD4^+^ T cell response. These results provide strong evidence that PYDG treatment increases specific T-cell mediated immune responses against HBV and accelerates HBV DNA clearance.

It is well established that predominantly noncytopathic mechanism contributes to HBV clearance [21]. This process is mediated primarily by inflammatory cytokines, including IFN-α/β, IFN-γ and TNF-α. Our study demonstrated that the levels of inflammatory cytokines, particularly IFN-γ and TNF-α in liver tissue interstitial fluid of HBV HDI mice were significantly increased following PYDG treatment. We also observed an increased immune response in PYDG-treated HBV specific T cells from acute or chronic Hepatitis patients (unpublished results). It is interesting to note that this enhanced immune response was not accompanied by the difference in the trend of HBsAg decrease between the PYDG and the control group. In this study, we observed that plasma DNA and HBcAg were reduced by the treatment, but not HBsAg, which is consistent to the clinical findings that serum HBsAg declines at a slow rate despite rapid and potent virologic suppression [22]. One possible explanation of this observation may be the minimal effect of the available treatment on the transcription/translation activity of the HBsAg gene or message and the presence of remaining empty subviral HBsAg particles in the circulation.

PYDG backbone composed of linear 1,3-linked D-glucose molecules (1,3-D-glucan), β-1, 6-linked side chains and three-dimensional helical structure, induces efficient stimulation of macrophages, DCs and lymphocytes [23]. The complement receptor 3 (CR3), lactosylceramide (LacCer), dectin-1 and TLR2 have been identified as glucan family receptors [23,24]. Binding of glucans to these receptors leads to the activation of macrophages, neutrophils, DCs, and some subsets of T cells to improve an immune response against bacterial, viral and fungal invasion and against tumors. In this study, we found that following oral administration, PYDG preferentially localized in the murine liver and promoted recruitment of DCs and other immune cells. Hence, our results support earlier observations demonstrating the capacity of PYDG in stimulating the maturation and activation of dendritic cells [25].

It has been shown that HBV infection leads to down-regulation of TLR2 expression and consequently impaired proinflammatory cytokines production in response to CpG stimulation [16,26]. Mousa et al. found that TLR2, expressed on activated T cells as a costimulatory receptor, directly contributes to the perpetuation and activation of long-term T cell memory [27]. It has been reported that the cooperation of TLR2 and dectin-1 could induce immune response in mouse and human primary monocytes and macrophages [28–30]. In our study, we confirmed the expression of dectin-1 receptor of T cell by flow cytometry and found that TLR2 expression on CD4+ and CD8+ T cells was strongly up-regulated in PYDG-treated mice. We speculated that PYDG engagement of dectin-1 receptor could induce T cells activation, which in turn would enhance TLR2 expression resulting in enlarged immune response. PYDG-mediated amplification of innate and adaptive immune responses through TLR2 and dectin-1 receptor may result in an enhanced anti-HBV immune response associated with HBV clearance.

Overall, our results provided evidence that PYDG effectively evoked the Th1 immune response against HBV in liver microenvironment. Importantly, PYDG-boosted HBV-specific T-cell immune responses in liver promoted HBV clearance without causing liver damage. Our findings strongly suggest that PYDG might be a promising immnuotherapeutic agent for the treatment of chronic HBV infection.

## Supporting Information

S1 Checklist ARRIVE Guidelines Checklist. (PDF)

S1 Fig HBsAg level detected in the HBV-HDI mice orally treated with PYDG. HBV-HDI mice were treated orally with PBS or 200μg/200μl PYDG daily for 9 weeks (n = 10). HBsAg level in plasma from mock and the treatment group was monitored over 9 weeks (*, p<0.05; **, p<0.01; ***, p<0.001).
(TIF)

S2 Fig PYDG distributed to the mouse liver and intestinal but not to kidney. DTAF-labeled PYDG was orally administered into C57BL/6 mice (n = 5) for three days. Mice were sacrificed and the liver, intestine and kidney were removed for frozen section preparation. The PYDG distribution was visualized under microscopy.
(TIF)
