# Supplementary figures and images for: Oral Administered Particulate Yeast-Derived Glucan Promotes Hepatitis B Virus Clearance in a Hydrodynamic Injection Mouse Model
Source: PLoS One. 2015 Apr 9;10(4):e0123559. doi: 10.1371/journal.pone.0123559 (PMC4391928; doi:10.1371/journal.pone.0123559)

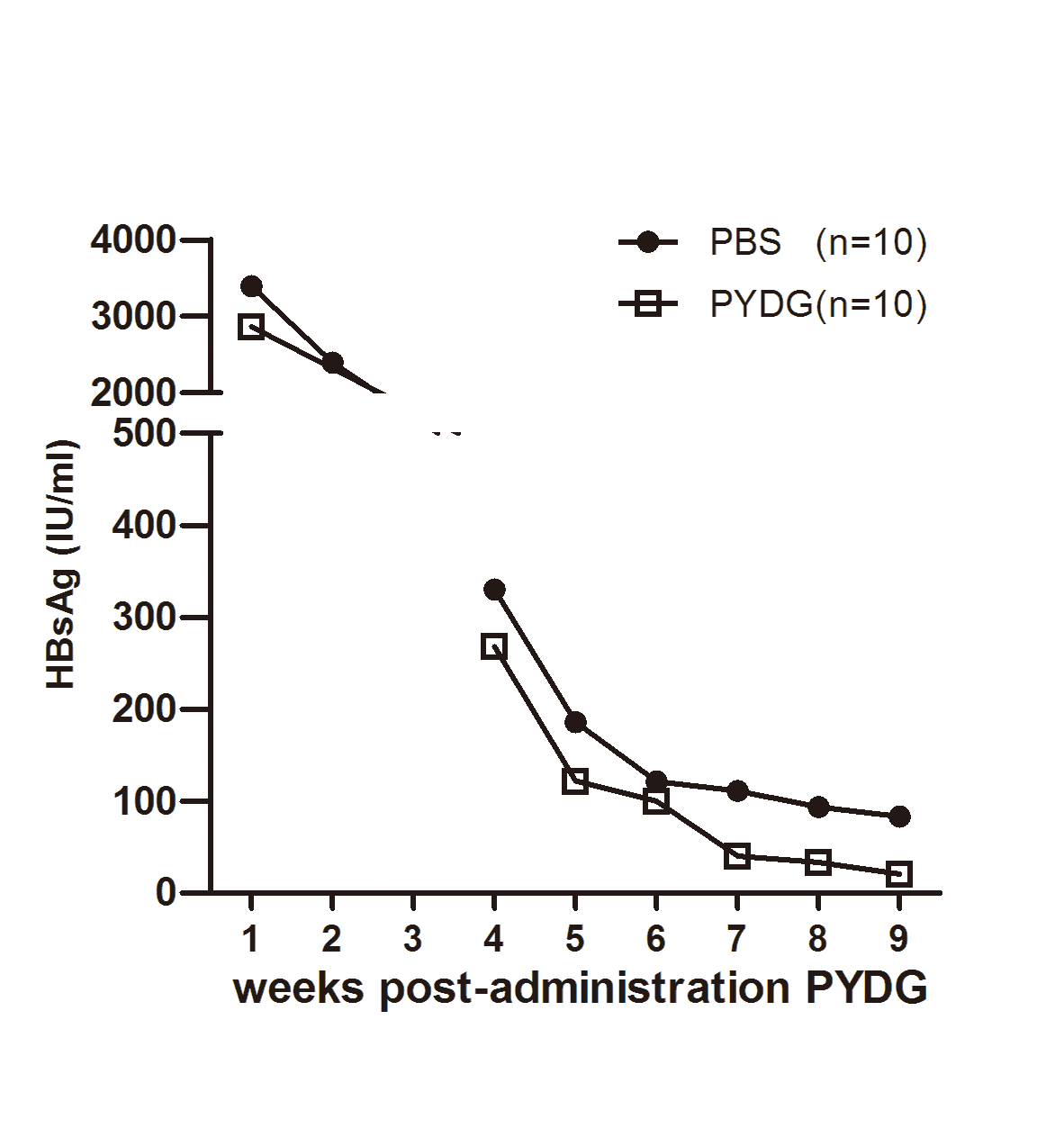

Supplement: S1 Fig — HBV-HDI mice were treated orally with PBS or 200μg/200μl PYDG daily for 9 weeks (n = 10). HBsAg level in plasma from mock and the treatment group was monitored over 9 weeks (*, p<0.05; **, p<0.01; ***, p<0.001). (TIF) [file pone.0123559.s002.tif]

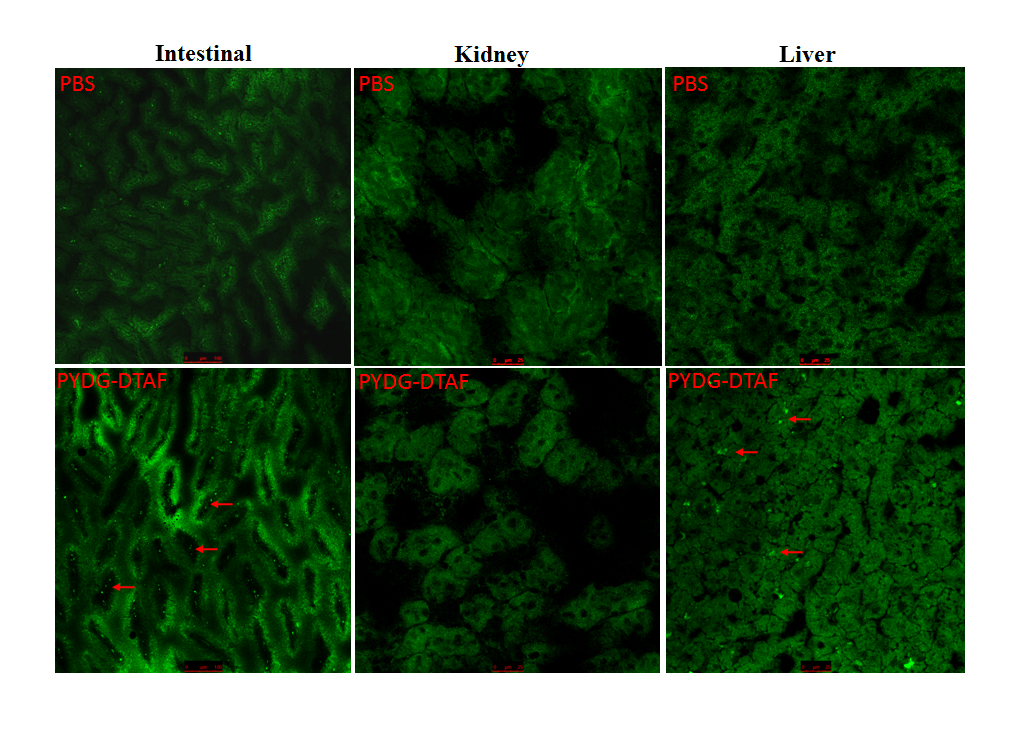

Supplement: S2 Fig — DTAF-labeled PYDG was orally administered into C57BL/6 mice (n = 5) for three days. Mice were sacrificed and the liver, intestine and kidney were removed for frozen section preparation. The PYDG distribution was visualized under microscopy. (TIF) [file pone.0123559.s003.tif]
